# Lifestyle risk factors for overweight and obesity among rural Indian adults: a community-based prospective cohort study

**DOI:** 10.1017/jns.2025.4

**Published:** 2025-02-11

**Authors:** Rajesh Kumar Rai, Sabri Bromage, Jan-Walter De Neve, Anamitra Barik

**Affiliations:** 1 Human Nutrition Unit, Institute of Nutrition, Mahidol University, Salaya, Nakhon Pathom, Thailand; 2 Society for Health and Demographic Surveillance, Suri, West Bengal, India; 3 Department of Global Health and Population, Harvard T H Chan School of Public Health, Boston, MA, USA; 4 Community Nutrition Unit, Institute of Nutrition, Mahidol University, Salaya, Nakhon Pathom, Thailand; 5 Department of Nutrition, Harvard T H Chan School of Public Health, Boston, MA, USA; 6 Heidelberg Institute of Global Health, Medical Faculty and University Hospital, University of Heidelberg, Heidelberg, Germany; 7 Division of Global Health Management and Policy, School of Public Health, San Diego State University, San Diego, CA, USA; 8 Suri District Hospital, Suri, West Bengal, India

**Keywords:** Diet, Obesity, Overweight, Physical activity, Substance use, BIRPOP, Birbhum Population Project, BMI, body mass index, CI, confidence interval, NCDs, non-communicable diseases, NPCDCS, National Programme for Prevention and Control of Cancer, Diabetes, Cardiovascular Diseases and Stroke, OBCs, other backward classes, RR, relative risk, SCs, scheduled castes, SHDS, Society for Health and Demographic Surveillance, STs, scheduled tribes, WHO, World Health Organization

## Abstract

India’s nutrition transition has led to an increased burden of overweight/obesity (body mass index of ≥23 kg/m^2^), driven by lifestyle factors like poor diet, inactivity, and substance use, prompting public health interventions. However, these interventions lack supporting evidence, especially in rural areas, hindering effective strategies for this population. To address this evidence gap, this study used cohort data (baseline: 2018–19, follow-up: 2022–23) from the Birbhum Population Project (West Bengal, India) to analyse lifestyle risk factors and their association with incidence and remission of overweight/obesity among adults aged ≥18 years (sample: 8,974). Modified Poisson regression model was employed to attain the study objective. From 2017–2018 to 2022–2023, the prevalence of overweight/obesity increased from 15.2% (95% CI: 14.1%–16.4%) to 21.0% (95% CI: 19.7%–22.3%) among men and from 24.1% (95% CI: 22.9%–25.2%) to 33.8% (95% CI: 32.5%–35.1%) among women. Overall, 23.0% (95% CI: 21.8%–24.3%) of adults experienced incidence of overweight/obesity, while 13.9% (95% CI: 12.4%–15.6%) experienced remission. Use of motor vehicles among unemployed participants was associated with incident overweight/obesity (relative risk or RR: 1.058; 95% CI: 1.023–1.095; P: 0.001). Vigorous activity at home (including gardening, yard work, and household chores) was linked to higher odds of recovering from overweight/obesity (RR: 1.065; 95% CI: 1.008–1.125; P: 0.025). Frequent tobacco use (often/daily vs. none) was inversely associated with remission of overweight-obesity (RR: 0.689; 95% CI: 0.484–0.980; P: 0.038), as was each 1 ml in alcohol consumption (RR: 0.995; 95% CI: 0.991–0.999; P: 0.022). Discouraging habitual motor vehicle use may help prevent overweight/obesity, while promoting home-based activities may aid remission, particularly for women who are at higher risk for overweight/obesity.

## Introduction

Nearly three-quarters of all deaths in low-and-middle-income countries are attributed to non-communicable diseases (NCDs), and India experiences the highest number of NCD-related mortality in the world.^(1)^ As estimated by the World Health Organization (WHO), NCDs accounted for a staggering 66% of total deaths in India in 2022, equivalent to approximately 6,047,000 deaths.^(1)^ Additionally, NCDs could detrimentally impact India’s economy, potentially reducing its gross domestic product by an estimated 0.8%.^(2)^ The aetiology of NCDs is multifaceted, encompassing behavioural factors (e.g. poor nutrition, physical inactivity, and substance use), environmental factors (e.g. unhealthy food environments, and limited healthcare access), and infections.^(3)^ However, the influence of these factors is significantly modulated by the socio-economic characteristics of the population. To tackle the increasing burden of NCDs in India,^(4)^ the Ministry of Health and Family Welfare, Government of India, developed a national NCD prevention strategy and renders technical and financial support to States and union territories under the National Programme for Prevention and Control of Cancer, Diabetes, Cardiovascular Diseases and Stroke (NPCDCS).^(5)^ Launched in 2010 within the National Health Mission, the NPCDCS implements community-level NCD prevention initiatives across all districts of India.

Modifiable lifestyle factors, such as diet, physical activity, and tobacco and alcohol use, are strongly implicated as risk factors for NCDs,^(1,6)^ partly due to their potential link with overweight and obesity, a key metabolic risk factor that can accelerate the development of NCDs. Mitigating the rising burden of overweight and obesity in India could significantly reduce the overall burden of NCDs.^(7,8)^ According to the 2019–2021 National Family Health Survey,^(9)^ the national prevalence of overweight and obesity is 22.9% among men and 24% among women aged 15–49 years. While the NPCDCS provides some guidelines for preventing overweight and obesity, its effectiveness remains to be established. Furthermore, the global literature on interventions targeting overweight and obesity predominantly focuses on children, particularly those of school age.^(10–12)^ Consequently, our understanding of potential strategies for preventing overweight and obesity and its determinants in adults is comparatively limited.

Although the burden of NCDs in India has grown substantially since 1990, the country lacks sufficiently detailed data on local NCD epidemiology to effectively inform research and policy formulation,^(13)^ including high-quality data on overweight and obesity and their determinants. Research on overweight and obesity in India has predominantly relied on cross-sectional datasets with a focus on socio-economic risk factors,^(8)^ resulting in a scarcity of information on the role of lifestyle factors.^(14–16)^ Additionally, operational definitions of lifestyle indicators, such as poor diet quality, inadequate physical activity, and excessive alcohol use, are often inconsistent. These challenges, along with concerns about the external validity of community-based studies in India, further limit our understanding of local overweight and obesity epidemiology.

A systematic review of dietary patterns in India^(17)^ concluded that the consumption of high-fat diets was associated with higher body mass index. Furthermore, a systematic review and dose-response meta-analysis^(18)^ estimated that alcohol use, particularly heavy drinking, increased the odds of overweight and obesity. While the effectiveness of physical activity interventions on weight loss and maintenance is unclear,^(19)^ systematic reviews^(20)^ convincingly demonstrated that increasing physical activity leads to weight loss. However, some argue^(21)^ that such interventions may be less effective at the population level and when not combined with dietary modification. Prior research in India has documented an inverse association between all forms of tobacco use and body mass index.^(22)^ However, global literature suggests a more complex relationship, with smoking potentially decreasing body weight in the short term but increasing it in the long term.^(23)^

The empirical evidence demonstrating the role of lifestyle risk factors in the development and mitigation of overweight and obesity in India is mixed and inconclusive. This is particularly true for rural populations, where research on the complex interplay of dietary habits, physical activity levels, tobacco and alcohol consumption patterns, and their impact on weight status remains limited. Further investigation using robust study designs and comprehensive data collection is needed to distinguish the specific influences of these lifestyle factors within the diverse socio-economic and cultural contexts of rural India. To address these evidence gaps, we analysed data from a prospective cohort study conducted as part of a large rural health and demographic surveillance system.^(24,25)^ Using data collected between 2017–2018 and 2022–2023, we estimated the prevalence, incidence and remission of overweight and obesity. We also examined relationships between key lifestyle risk factors (measured at baseline) and both the incidence and remission of overweight and obesity.

## Methods

### Data and study population

Data used for this study were collected as part of the Birbhum Population Project (BIRPOP), a Health and Demographic Surveillance System located in the Birbhum district of the state of West Bengal, India.^(24,25)^ Established by the Department of Health and Family Welfare, Government of West Bengal in 2008, BIRPOP functions under the ambit of the Society for Health and Demographic Surveillance (SHDS). Largely rural, the district of Birbhum is classified as one of the “backward” districts by the Government of India, and BIRPOP-SHDS covers four administrative blocks (Suri 1, Sainthia, Rajnagar, and Mohammad Bazar) which represent nearly 16% of the district’s population. At its inception, a cohort of 13,053 self-weighted households (59,395 individuals, 51.1% male and 48.9% female) was randomly sampled using the 2001 census sampling frame and accounting for a 10% expected non-response rate.^(24,25)^ BIRPOP-SHDS conducts routine surveys (collecting data on vital events; antenatal, natal and postnatal tracking; and verbal autopsy), as well as focused surveys and interventions related to socio-economics, nutrition, NCDs and other areas. Details on sampling procedures and surveys implemented in BIRPOP-SHDS can be obtained from its published reports.^(8,24–27)^

During 2017–2018, BIRPOP-SHDS undertook a survey to understand the role of modifiable determinants of body mass index^(28)^ among rural Indians aged ≥18 years (sample: 11,399). Information on demographic and socio-economic characteristics, dietary practices, physical activity, and alcohol and tobacco use were collected and participant’s height and weight were measured. A follow-up survey, conducted in 2022–2023, measured height and weight among 8,974 (78.7%) of those in the initial survey. Attrition of 2,425 individuals was attributed to permanent/ temporary migration (38.1%), absenteeism (34.6%), pregnancy (17.3%) and death (10%).

### Incidence and remission of overweight including obesity

**Figure 1.**
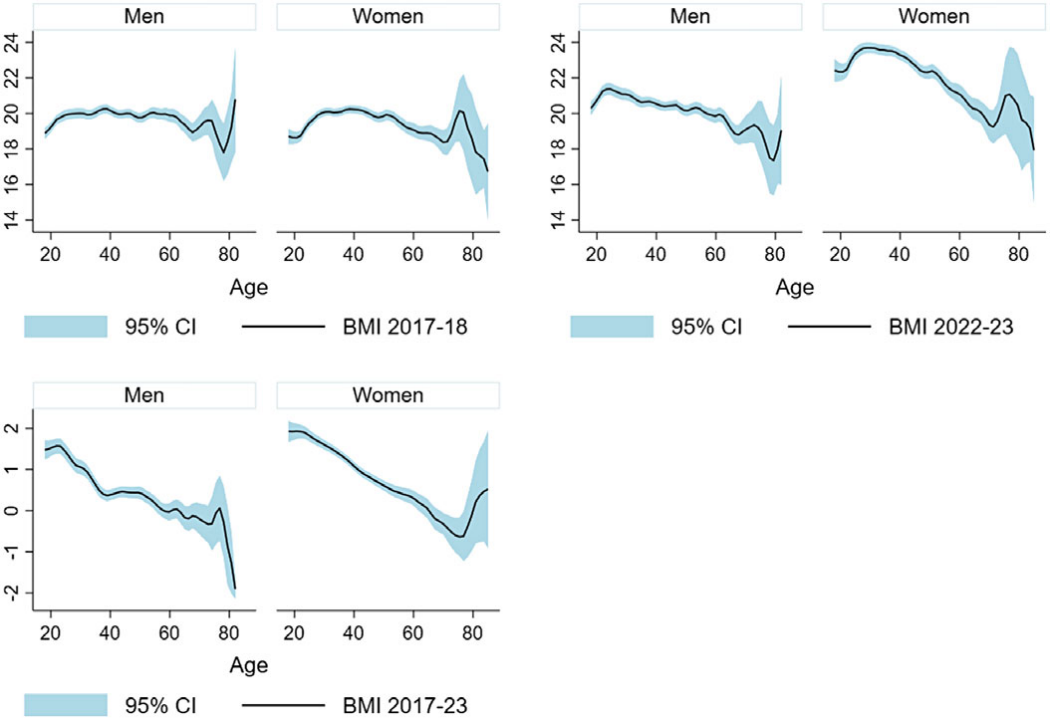
Local polynomial-smoothed plots of mean (with 95% confidence interval or CI) body mass index (BMI) during 2017–18 (top left), 2022–2023 (top right), and change in BMI from 2017–2018 to 2022–2023 (bottom left) by age and gender.

To calculate body mass index (BMI, an individual’s weight in kilograms divided by their height in metres squared), a standardised height and weight measurement protocol and inclusion and exclusion criteria were followed to obtain height and weight data in the study population.^(29)^ Height was measured using a stadiometer (Bioplus Stature Meter, model IND/09/2005/815) and weight was measured using a certified electronic scale (Omron HN-283). These measurements were taken by trained surveyors of BIRPOP-SHDS. “Overweight/obesity” was defined as a BMI of ≥23 kg/m^2^ according to WHO guidelines developed for Asian Indians.^(30)^ Following definitions used in an earlier study with the BIRPOP-SHDS population,^(29)^ incidence of overweight/obesity was defined as a transition from normal BMI (18.5–22.9 kg/m^2^) in 2017–2018 to overweight/obesity in 2022–2023, while remission was defined as a transition from overweight/obesity in 2017–2018 to normal BMI in 2022–2023. Smoothed plots (graphically presented using local polynomial smoothing) of mean BMI during 2017–2018 and 2022–2023 and change from 2017–2018 to 2022–2023 by gender an age are presented in Figure 1. At baseline (2017–2018), a mean BMI of approximately 20.7 kg/m^2^ was observed among men from their mid-twenties to late fifties. In contrast, mean BMI among women peaked at 40 years (nearly 21.3 kg/m^2^) and decreased thereafter. The change in BMI from 2017–2018 to 2022–2023 was highest among men and women aged 20 years at baseline (0.5 kg/m^2^) and lowest around 80 years old (nearly –0.5 kg/m^2^).

### Lifestyle risk factors

Lifestyle risk factors for overweight/obesity were represented by indicators on dietary habits, physical activity, alcohol consumption, and tobacco use. Dietary habits were assessed using food group-specific frequency options,capturing consumption of meat, milk, lentils, fruits, vegetables, and added salt. Consumption of four categories of processed or prepared foods (potato chips/wafers, other snacks, *paratha/luchi/puri*, and *biriyani/fried rice*) and food consumed outside the home were also assessed. Physical activity was measured as the number of days per week that participants engaged in at least 10 minutes of both vigorous and moderate physical activity during both leisure and non-leisure time, walking (assessed among both employed and unemployed participants), and use of different transportation modalities (unemployed participants only). Participants were asked about their alcohol consumption - those who reported ever consuming alcohol were then asked about current consumption (defined as any consumption in the past 30 days) and their average daily volume of alcohol consumed. Frequency of smoking and smokeless tobacco use were also assessed, and participants were categorised as ‘often/daily smokers/users’ and ‘non-smokers/users’.

### Statistical approach

The prevalence of overweight/obesity, with associated 95% confidence intervals (CIs), was calculated for both men and women in 2017–2018 and 2022–2023. Prevalence was examined within strata defined by key background characteristics and lifestyle risk factors. Information on background characteristics included each participant’s age, years of schooling, occupation, religion, social group, wealth quintile, mass media exposure, distance from their house to the nearest healthcare facility, and health insurance coverage. Social groups, namely Scheduled Castes (SCs), Scheduled Tribes (STs), Other Backward Classes (OBCs), and Others, were defined according to the Constitution of India. The Constitution recognises SCs, STs, and OBCs as economically disadvantaged compared to the ‘Others’ group.^(29)^ Information on household goods and durables was used to derive a wealth index using principal component analysis.^(31)^ To measure mass media exposure, respondents were asked how often they read newspapers, listen to radio, or watch television (almost every day or high exposure, once or more per month medium exposure, or once or less per three months or low exposure).^(32)^ Modified Poisson regression models with robust error variance^(33)^ were used to estimate relative risks (RRs) with 95% CIs of overweight/obesity incidence and remission associated with each lifestyle risk factor, adjusting for potential confounders. The statistical software Stata version 18^(34)^ was used for all analyses and statistical significance was defined based on two-tailed P-values <0.05.

## Results

From 2017–2018 to 2022–2023, the prevalence of overweight/obesity increased by nearly six percentage points among men (from 15.2% (95% CI: 14.1%–16.4%) to 21.0% (95% CI: 19.7%–22.3%)) and nearly ten percentage points among women (from 24.1% (95% CI: 22.9%–25.2%) to 33.8% (95% CI: 32.5%–35.1%)) (Table 1). Among both men and women, alcohol users had a lower prevalence of overweight/obesity than non-users, while those who used tobacco (including both smoked and smokeless tobacco) had a higher prevalence of overweight/obesity than non-users. Overweight/obesity was more prevalent among both the wealthiest individuals (compared to the poorest) and those with high mass media exposure (compared to those with low exposure).

**Table 1.** Prevalence (%) of overweight/obesity by key lifestyle factors and background characteristics among men and women, 2017–2018 and 2022–2023

	n		2017–2018		2022–2023	
	Men	Women	Men% (95% CI)	Women% (95% CI)	Men% (95% CI)	Women% (95% CI)
Dietary habits						
*Consumption of meat*						
Not at all /<2 times/month	1,841	2,596	12,4 (11.0–14.0)	18.5 (17.0–20.0)	17.9 (16.2–19.7)	27.2 (25.5–28.9)
2+ times/month	1,872	2,665	18.0 (16.3–19.8)	29.5 (27.8–31.3)	24.0 (22.2–26.0)	40.3 (38.4–42.1)
*Consumption of milk*						
Not at all/rarely	1,975	3,086	10.5 (9.3–12.0)	19.5 (18.1–20.9)	15.2 (13.7–16.9)	29.2 (27.6–30.8)
≤4 days/month	475	588	14.1 (11.3–17.5)	23.5 (20.2–27.1)	22.5 (19.0–26.5)	34.2 (30.5–38.1)
Almost everyday	1,263	1,587	23.0 (20.7–25.4)	33.2 (30.9–35.6)	29.4 (26.9–31.9)	42.7 (40.2–45.1)
*Consumption of lentils*						
Not at all/rarely/≤4 days/month	2,010	2,876	10.6 (9.3–12.0)	18.3 (16.9–19.8)	15.6 (14.1–17.3)	27.5 (25.9–29.2)
Almost everyday	1,703	2,385	20.7 (18.9–22.7)	31.0 (29.2–32.9)	27.3 (25.2–29.5)	41.3 (39.4–43.3)
*Consumption of fruit*						
Not at all in past 7 days	2,426	3,465	12.1 (10.9–13.5)	19.7 (18.4–21.0)	17.1 (15.7–18.7)	29.7 (28.2–31.2)
1–2 times/day in past 7 days	769	1,081	17.4 (14.9–20.3)	27.0 (24.4–29.7)	25.5 (22.5–28.7)	36.4 (33.5–39.3)
3+ times/day in past 7 days	518	715	26.4 (22.8–30.4)	41.0 (37.4–44.6)	32.4 (28.5–36.6)	49.8 (46.1–53.5)
*Consumption of vegetables*						
<7 days in past 7 days	606	906	10.9 (8.6–13.6)	19.3 (16.9–22.0)	16.7 (13.9–19.9)	28.6 (25.7–31.6)
Every day in past 7 days	3,107	4,355	16.1 (14.8–17.4)	25.1 (23.8–26.4)	21.8 (20.4–23.3)	34.9 (33.5–36.3)
*Added salt to food before eating*						
Sometimes/rarely/never	1,417	1,943	11.6 (10.0–13.3)	19.4 (17.7–21.2)	18.1 (16.1–20.2)	30.4 (28.4–32.4)
Always/often	2,296	3,318	17.5 (16.0–19.1)	26.8 (25.3–28.3)	22.8 (21.1–24.5)	35.8 (34.2–37.5)
*Processed and prepared foods*						
Potato chips/wafers						
Some days/once/never in past 7 days	3,247	4,599	15.2 (14.0–16.5)	24.0 (22.8–25.3)	20.4 (19.0–21.8)	33.5 (32.2–34.9)
Everyday/most days in past 7 days	466	662	15.5 (12.4–19.0)	24.3 (21.2–27.7)	25.1 (21.4–29.3)	35.6 (32.1–39.4)
Other snacks						
Some days/once/never in past 7 days	3,103	4,670	14.4 (13.2–15.7)	23.3 (22.1–24.5)	20.1 (18.7–21.5)	32.9 (31.6–34.3)
Everyday/most days in past 7 days	610	591	19.2 (16.2–22.5)	30.1 (26.5–33.9)	25.6 (22.3–29.2)	40.9 (37.0–45.0)
*Paratha/Luchi/Puri*						
Once/never in past 7 days	3,150	4,602	13.7 (12.5–14.9)	22.8 (21.6–24.0)	19.1 (17.8–20.5)	32.7 (31.4–34.1)
Some days/everyday/most days in past 7 days	563	659	23.8 (20.5–27.5)	33.2 (29.7–36.9)	31.4 (27.7–35.4)	41.4 (37.7–45.2)
* Biriyani/*Fried rice						
Never in past 7 days	3,429	4,902	14.4 (13.2–15.6)	23.3 (22.1–24.5)	20.0 (18.7–21.4)	32.5 (31.2–33.8)
At least once in past 7 days	284	359	25.4 (20.6–30.7)	35.1 (30.3–40.2)	32.7 (27.5–38.4)	51.5 (46.4–56.7)
*Food consumed outside home*						
Never in past 7 days	3,020	4,793	14.8 (13.5–16.1)	24.1 (22.9–25.3)	19.8 (18.4–21.3)	33.6 (32.3–34.9)
≥1 day in past 7 days	693	468	17.2 (14.5–20.2)	23.7 (20.1–27.8)	26.0 (22.8–29.4)	35.9 (31.7–40.4)
Alcohol use						
Ever consumed						
No	2,541	4,999	17.7 (16.3–19.2)	24.9 (23.7–26.1)	23.8 (22.2–25.5)	34.8 (33.5–36.2)
Yes	1,172	262	9.8 (8.2–11.7)	8.0 (5.3–12.0)	14.8 (12.8–16.9)	14.1 (10.4–18.9)
Current user (in past 30 days)						
No	2,869	5,077	16.8 (15.5–18.2)	24.6 (23.5–25.8)	23.0 (21.5–24.5)	34.5 (33.3–35.9)
Yes	844	184	9.7 (7.9–11.9)	8.2 (5.0–13.1)	14.2 (12.0–16.7)	13.0 (8.9–18.7)
Mean alcohol consumption (in ml)	3,713	5,261				
Physical activity^*^						
Employed persons only						
Vigorous activity	3,713	5,261	–	–	–	–
Moderate-intensity activity	3,713	5,261	–	–	–	–
Walking	3,713	5,261	–	–	–	–
Unemployed persons only						
Motorcycle/scooter/auto-rickshaw/train/bus/car	3,713	5,261	–	–	–	–
Bicycle	3,713	5,261	–	–	–	–
Walking	3,713	5,261	–	–	–	–
Vigorous physical activity inside the house (garden/yard/household work)						
Moderate-intensity activity inside the house	3,713	5,261	–	–	–	–
Leisure time (employed and unemployed persons)			–	–	–	–
Vigorous physical activity	3,713	5,261	–	–	–	–
Moderate-intensity activity	3,713	5,261	–	–	–	–
Tobacco use						
Smoking (bidi/cigarette/rolled cigar/others)						
Often/daily	1,923	124	12.5 (11.1–14.1)	7.3 (3.8–13.4)	17.2 (15.5–18.9)	11.3 (6.8–18.2)
Non-smoker	1,790	5,137	18.1 (16.4–20.0)	24.5 (23.3–25.7)	25.1 (23.1–27.1)	34.3 (33.1–35.6)
Smokeless tobacco use (gutka/snuff/chewing tobacco/betel quid/others)						
Often/daily	1,110	1,036	14.7 (12.7–16.9)	18.0 (15.7–20.4)	20.5 (18.3–23.0)	25.0 (22.5–27.7)
Non-user	2,603	4,225	15.4 (14.1–16.9)	25.6 (24.3–26.9)	21.2 (19.6–22.8)	36.0 (34.5–37.4)
Age						
18–24 years	484	373	10.5 (8.1–13.6)	12.1 (9.1–15.8)	24.0 (20.4–28.0)	27.9 (23.6–32.7)
25–34 years	650	1,432	15.8 (13.2–18.9)	24.8 (22.6–27.1)	25.7 (22.5–29.2)	39.0 (36.5–41.5)
35–44 years	943	1,506	16.2 (14.0–18.7)	27.6 (25.4–29.9)	21.4 (18.9–24.2)	38.6 (36.2–41.1)
≥45 years	1,636	1,950	15.8 (14.1–17.6)	23.1 (21.3–25.1)	18.0 (16.2–19.9)	27.4 (25.5–29.5)
Years of schooling	3,713	5,261	–	–	–	–
Occupation						
Unemployed	368	4,157	13.9 (10.7–17.8)	25.8 (24.5–27.1)	18.5 (14.8–22.8)	35.7 (34.3–37.2)
Labour (agricultural and non-agricultural)	1,841	822	9.9 (8.6–11.3)	11.9 (9.9–14.3)	16.2 (14.6–17.9)	22.1 (19.4–25.1)
In service/self-employed	1,504	282	22.1 (20.0–24.2)	34.4 (29.1–40.1)	27.5 (25.3–29.8)	39.0 (33.5–44.8)
Religion						
Hinduism	2,651	3,618	14.3 (13.1–15.7)	20.7 (19.4–22.0)	19.2 (17.8–20.8)	28.6 (27.2–30.1)
Islam/others	1,062	1,643	17.4 (15.3–19.8)	31.5 (29.3–33.8)	25.3 (22.8–28.0)	45.2 (42.8–47.6)
Social group						
Others	1,484	2,291	20.6 (18.6–22.7)	31.2 (29.3–33.1)	26.1 (24.0–28.4)	41.9 (39.9–43.9)
OBCs	566	680	15.9 (13.1–19.2)	32.2 (28.8–35.8)	23.5 (20.2–27.2)	41.9 (38.3–45.7)
SCs/STs	1,663	2,290	10.2 (8.9–11.8)	14.5 (13.1–16.0)	15.5 (13.9–17.3)	23.3 (21.6–25.1)
Wealth quintile						
Poorest	709	1,178	5.5 (4.0–7.4)	12.4 (10.6–14.4)	9.9 (7.9–12.3)	20.0 (17.8–22.4)
Poorer	713	1,075	7.2 (5.5–9.3)	15.5 (13.5–17.8)	13.3 (11.0–16.0)	26.9 (24.3–29.6)
Middle	749	983	11.3 (9.3–13.8)	21.3 (18.8–23.9)	19.0 (16.3–21.9)	32.2 (29.4–35.2)
Richer	833	1,157	17.9 (15.4–20.6)	32.0 (29.4–34.7)	23.5 (20.8–26.5)	41.8 (39.0–44.7)
Richest	709	868	34.0 (30.6–37.6)	43.1 (39.8–46.4)	38.9 (35.4–42.6)	52.1 (48.7–55.4)
Mass media exposure						
High	2,681	3,602	18.5 (17.1–20.0)	28.0 (26.5–29.5)	24.5 (22.9–26.1)	38.0 (36.4–39.6)
Medium	331	475	7.6 (5.2–10.9)	18.7 (15.5–22.5)	12.7 (9.5–16.7)	29.9 (25.9–34.2)
Low	701	1,184	6.3 (4.7–8.3)	14.3 (12.4–16.4)	11.6 (9.4–14.1)	22.6 (20.3–25.0)
Distance from home to nearest healthcare facility						
<5 km	1,828	2,685	16.6 (15.0–18.4)	24.7 (23.1–26.4)	22.0 (20.2–23.9)	33.4 (31.7–35.3)
5–10 km	1,751	2,367	13.8 (12.3–15.5)	23.4 (21.8–25.2)	20.5 (18.7–22.5)	34.9 (33.0–36.8)
>10 km	134	209	14.2 (9.2–21.2)	22.5 (17.3–28.7)	13.4 (8.6–20.3)	25.8 (20.3–32.2)
Health insurance						
Yes	2,045	3,002	12.2 (10.8–13.7)	21.2 (19.8–22.7)	17.9 (16.3–19.6)	31.7 (30.1–33.4)
No	1,668	2,259	18.9 (17.1–20.9)	27.8 (26.0–29.7)	24.8 (22.7–26.9)	36.5 (34.6–38.5)
Total	3,713	5,261	15.2 (14.1–16.4)	24.1 (22.9–25.2)	21.0 (19.7–22.3)	33.8 (32.5–35.1)

n, sample; CI, confidence interval; km, kilometre; ml, millilitre; OBCs, other backward classes; SCs, scheduled castes; STs, scheduled tribes.

– : percentage estimates were not calculated as the covariates are continuous variables.

* Recorded as number of days per week engaged in at least 10 minutes of each activity category.

Table 2 presents the incidence of overweight/obesity and its associated lifestyle factors. Among a total of 4,056 participants with normal BMI in 2017–2018, 23.0% (95% CI: 21.8%–24.3%) developed overweight/obesity by 2022–2023. Multivariable models revealed a positive association between the use of a motorised vehicle (motorcycle, scooter, auto-rickshaw, car, etc.) among unemployed study participants and the development of overweight/obesity (RR: 1.058; 95% CI: 1.023–1.095; P: 0.001). Among 1,831 participants with overweight/obesity in 2017–2018, 13.9% (95% CI: 12.4%–15.6%) experienced remission as measured during 2022–2023 (Table 3). Each 1 ml in alcohol consumption was also inversely associated with remission (RR: 0.995; 95% CI: 0.991–0.999; P: 0.022). Among unemployed adults, vigorous home-based physical activity (including gardening, yard work, and household chores) was linked to higher odds of recovering from overweight/obesity (RR: 1.065; 95% CI: 1.008–1.125; P: 0.025). Compared to those who used tobacco often/daily, non-users were less likely to experience remission from overweight/obesity (RR: 0.689; 95% CI: 0.484–0.980; P: 0.038).

**Table 2.** Incidence of overweight/obesity from 2017–2018 to 2022–2023 and its associations with key lifestyle risk factors

	N	% (95% CI)	RR^*^	95% CI	P
Dietary habits					
*Consumption of meat*					
Not at all /<2 times/month	2,005	20.4 (18.6–22.2)	1.00 (ref.)		
2+ times/month	2,051	25.6 (23.8–27.6)	1.024	0.900–1.165	0.718
*Consumption of milk*					
Not at all/rarely	2,258	21.7 (20.0–23.4)	1.00 (ref.)		
≤4 days/month	508	24.8 (21.2–28.8)	1.058	0.889–1.259	0.526
Almost everyday	1,290	24.7 (22.4–27.1)	1.018	0.880–1.178	0.807
*Consumption of lentils*					
Not at all/rarely/≤4 days/month	2,175	21.0 (19.3–22.7)	1.00 (ref.)		
Almost everyday	1,881	25.4 (23.5–27.4)	1.081	0.953–1.227	0.224
*Consumption of fruit*					
Not at all in past 7 days	2,616	22.4 (20.8–24.0)	1.00 (ref.)		
1–2 times/day in past 7 days	908	23.0 (20.4–25.9)	0.944	0.818–1.088	0.425
3+ times/day in past 7 days	532	26.3 (22.7–30.2)	0.974	0.819–1.158	0.766
*Consumption of vegetables*					
<7 days in past 7 days	649	22.3 (19.3–25.7)	1.00 (ref.)		
Every day in past 7 days	3,407	23.2 (21.8–24.6)	0.952	0.815–1.112	0.533
*Added salt to food before eating*					
Sometimes/rarely/never	1,541	22.9 (20.9–25.1)	1.00 (ref.)		
Always/often	2,515	23.1 (21.5–24.8)	1.018	0.906–1.144	0.764
*Processed and prepared foods*					
Potato chips/wafers					
Some days/once/never in past 7 days	3,524	22.8 (21.5–24.3)	1.00 (ref.)		
Everyday/most days in past 7 days	532	24.2 (20.8–28.1)	1.049	0.885–1.245	0.580
Other snacks					
Some days/once/never in past 7 days	3,515	22.8 (21.4–24.2)	1.00 (ref.)		
Everyday/most days in past 7 days	541	24.6 (21.1–28.4)	0.938	0.793–1.110	0.456
* Paratha/Luchi/Puri*					
Once/never in past 7 days	3,477	22.6 (21.3–24.1)	1.00 (ref.)		
Some days/everyday/most days in past 7 days	579	25.4 (22.0–29.1)	1.023	0.871–1.202	0.782
* Biriyani/*Fried rice					
Never in past 7 days	3,737	22.4 (21.1–23.8)	1.00 (ref.)		
At least once in past 7 days	319	30.1 (25.3–35.4)	1.112	0.921–1.342	0.269
*Food consumed outside home*					
Never in past 7 days	3,514	22.7 (21.4–24.1)	1.00 (ref.)		
≥1 day in past 7 days	542	25.1 (21.6–28.9)	1.123	0.954–1.320	0.163
Alcohol use					
Ever consumed					
No	3,396	24.7 (23.3–26.2)	1.00 (ref.)		
Yes	660	14.2 (11.8–17.1)	1.073	0.773–1.491	0.673
Current user (in past 30 days)					
No	3,581	24.4 (23.0–25.8)	1.00 (ref.)		
Yes	475	12.8 (10.1–16.2)	0.989	0.612–1.598	0.965
Mean alcohol consumption (in ml)	4,056	–	0.999	0.998–1.000	0.116
Physical activity					
Employed persons only					
Vigorous activity	4,056	–	1.018	0.958–1.082	0.558
Moderate-intensity activity	4,056	–	0.962	0.916–1.009	0.114
Walking	4,056	–	1.035	0.984–1.089	0.183
Unemployed persons only					
Motorcycle/scooter/auto-rickshaw/train/bus/car	4,056	–	1.058	1.023–1.095	0.001
Bicycle	4,056	–	1.030	0.999–1.061	0.061
Walking	4,056	–	0.989	0.969–1.010	0.303
Vigorous physical activity inside the house (garden/yard/household work)	4,056	–	1.025	0.999–1.051	0.055
Moderate-intensity activity inside the house	4,056	–	1.010	0.984–1.037	0.438
Leisure time (employed and unemployed persons)					
Vigorous physical activity	4,056	–	0.943	0.835–1.065	0.343
Moderate-intensity activity	4,056	–	0.993	0.939–1.050	0.801
**Tobacco use**					
Smoking					
Smoking (bidi/cigarette/rolled cigar/others)					
Often/daily	940	15.1 (13.0–17.5)	1.00 (ref.)		
Non-smoker	3,116	25.4 (23.9–27.0)	1.170	0.947–1.446	0.145
Smokeless tobacco use (gutka/snuff/chewing tobacco/betel quid/others)					
Often/daily	977	18.6 (16.3–21.2)	1.00 (ref.)		
Non-user	3,079	24.4 (22.9–26.0)	1.131	0.974–1.314	0.106
Total	4,056	23.0 (21.8–24.3)			

n, sample; CI, confidence interval; ml, millilitre; RR, relative risk; (ref.), referent.

* Predictors are adjusted for age, gender, years of schooling, occupation, religion, social group, wealth quintile, mass media exposure, distance to nearest health facility, and health insurance.

– : percentage estimates were not calculated as the covariates are continuous variables.

**Table 3.** Remission of overweight/obesity from 2017–2018 to 2022–2023, and its associations with key lifestyle risk factors

	N	% (95% CI)	RR^*^	95% CI	P
Dietary habits					
*Consumption of meat*					
Not at all /<2 times/month	708	15.8 (13.3–18.7)	1.00 (ref.)		
2+ times/month	1,123	12.7 (10.9–14.8)	1.070	0.831–1.378	0.600
*Consumption of milk*					
Not at all/rarely	809	14.8 (12.5–17.5)	1.00 (ref.)		
≤4 days/month	205	13.7 (9.6–19.1)	0.873	0.598–1.277	0.485
Almost everyday	817	13.1 (10.9–15.6)	0.791	0.597–1.050	0.104
*Consumption of lentils*					
Not at all/rarely/≤4 days/month	738	15.6 (13.1–18.4)	1.00 (ref.)		
Almost everyday	1,093	12.8 (11.0–14.9)	0.854	0.657–1.111	0.241
*Consumption of fruit*					
Not at all in past 7 days	975	15.2 (13.1–17.6)	1.00 (ref.)		
1–2 times/day in past 7 days	426	12.2 (9.4–15.7)	0.891	0.657–1.208	0.457
3+ times/day in past 7 days	430	12.8 (9.9–16.3)	0.923	0.664–1.284	0.636
*Consumption of vegetables*					
<7 days in past 7 days	241	12.9 (9.2–17.7)	1.00 (ref.)		
Every day in past 7 days	1,590	14.1 (12.5–15.9)	1.122	0.784–1.607	0.530
*Added salt to food before eating*					
Sometimes/rarely/never	541	12.4 (9.9–15.4)	1.00 (ref.)		
Always/often	1,290	14.6 (12.7–16.6)	1.182	0.899–1.553	0.231
*Processed and prepared foods*					
Potato chips/wafers					
Some days/once/never in past 7 days	1,598	14.5 (12.8–16.3)	1.00 (ref.)		
Everyday/most days in past 7 days	233	10.3 (7.0–14.9)	0.759	0.495–1.165	0.207
Other snacks					
Some days/once/never in past 7 days	1,536	14.2 (12.5–16.0)	1.00 (ref.)		
Everyday/most days in past 7 days	295	12.5 (9.2–16.8)	1.013	0.723–1.419	0.941
* Paratha/Luchi/Puri*					
Once/never in past 7 days	1,478	13.6 (11.9–15.4)	1.00 (ref.)		
Some days/everyday/most days in past 7 days	353	15.3 (11.9–19.4)	1.229	0.917–1.648	0.168
* Biriyani/*Fried rice					
Never in past 7 days	1,633	14.3 (12.7–16.1)	1.00 (ref.)		
At least once in past 7 days	198	11.1 (7.4–16.3)	0.955	0.624–1.463	0.833
*Food consumed outside home*					
Never in past 7 days	1,601	14.4 (12.7–16.2)	1.00 (ref.)		
≥1 day in past 7 days	230	10.9 (7.4–15.6)	0.718	0.480–1.073	0.106
Alcohol use					
Ever consumed					
No	1,695	13.6 (12.1–15.3)	1.00 (ref.)		
Yes	136	17.6 (12.1–25.0)	0.742	0.375–1.469	0.392
Current user (in past 30 days)					
No	1,734	13.7 (12.2–15.4)	1.00 (ref.)		
Yes	97	17.5 (11.2–26.5)	2.441	0.823–7.241	0.108
Mean alcohol consumption (in ml)	1,831	–	0.995	0.991–0.999	0.022
Physical activity					
Employed persons only					
Vigorous activity	1,831	–	1.018	0.898–1.153	0.781
Moderate-intensity activity	1,831	–	0.971	0.882–1.069	0.550
Walking	1,831	–	1.054	0.947–1.174	0.335
Unemployed persons only					
Motorcycle/scooter/auto-rickshaw/train/bus/car	1,831	–	0.989	0.930–1.053	0.738
Bicycle	1,831	–	1.018	0.963–1.076	0.533
Walking	1,831	–	0.999	0.960–1.040	0.959
Vigorous physical activity inside the house (garden/yard/household work)	1,831	–	1.065	1.008–1.125	0.025
Moderate-intensity activity inside the house	1,831	–	1.020	0.970–1.072	0.437
Leisure time (employed and unemployed persons)					
Vigorous physical activity	1,831	–	0.912	0.699–1.189	0.496
Moderate-intensity activity	1,831	–	1.034	0.960–1.113	0.376
Tobacco use					
Smoking					
Smoking (bidi/cigarette/rolled cigar/others)					
Often/daily	250	21.6 (16.9–27.1)	1.00 (ref.)		
Non-smoker	1,581	12.7 (11.2–14.4)	0.689	0.484–0.980	0.038
Smokeless tobacco use (gutka/snuff/chewing tobacco/betel quid/others)					
Often/daily	349	14.3 (11.0–18.4)	1.00 (ref.)		
Non-user	1,482	13.8 (12.2–15.7)	1.067	0.787–1.447	0.676
Total	1,831	13.9 (12.4–15.6)			

n, sample; CI, confidence interval; ml, millilitre; RR, relative risk; (ref.), referent.

* Predictors are adjusted for age, gender, years of schooling, occupation, religion, social group, wealth quintile, mass media exposure, distance to nearest health facility, and health insurance.

– : percentage estimates were not calculated as the covariates are continuous variables.

## Discussion

Using prospective cohort data from a health and demographic surveillance system in West Bengal, India, this study examined associations between key lifestyle risk factors (dietary habits, physical activity, and alcohol and tobacco use) measured at baseline and subsequent incidence and remission of overweight/obesity among rural Indian men and women between 2017–2018 and 2022–2023.

This study observed a considerably higher prevalence and increase in the prevalence of overweight and obesity in women than in men, consistent with prior research^(29)^ conducted in the BIRPOP-SHDS cohort (baseline survey: 2008; follow-up survey: 2017). These findings may be partly explained by biological factors such as age-related weight gain and hormonal and pregnancy-related effects among women.^(35)^ However, sociocultural factors are also crucial to consider. Women in rural India often face constraints on their mobility and autonomy due to traditional gender roles and limited social support.^(36)^ This can restrict their opportunities for engaging in physical activity. Additionally, in rural India there often exists a division of labour wherein women typically engage in less physically demanding tasks than men, further contributing to lower activity levels.^(37)^

This study revealed a link between increased use of motorised transport and a higher likelihood of developing overweight/obesity. These findings are consistent with prior research in India, which utilised data from the National Family Health Survey (2005–2006 and 2015–2016) and identified a positive association between household motor vehicle ownership and obesity, even after accounting for socio-economic factors.^(38)^ Similar results have been reported in studies conducted in Bangladesh.^(39)^ However, this study makes a unique contribution to the existing literature in that while earlier research focused on vehicle ownership at the household level, this study examined individual vehicle use.

This study provides evidence that engaging in vigorous physical activity at home – such as gardening, yard work, and household chores - was conducive to remission from overweight/obesity. In addition to increasing energy expenditure, engaging in physical activity may improve metabolic health, further supporting weight management and overall health. These findings are encouraging, especially for women in India who may have limited opportunities to participate in structured exercise programmes. Engaging in physical activity at home, like household chores, gardening, or playing with children, could offer a practical and accessible way to increase their energy expenditure and manage weight.^(40)^

Alcohol use (expressed as a continuous variable) and abstinence from tobacco use were inversely associated with remission from overweight-obesity. An earlier study reported that all forms of tobacco use are associated with low BMI in India,^(22)^ while a study conducted in the BIRPOP-SHDS cohort found that remission from overweight/obesity was less likely among alcohol users.^(29)^ These findings may be attributed in part to lower levels of physical activity among alcohol and tobacco users, in addition to appetite-suppressing effects of nicotine and the obesogenic effects of alcohol, respectively.^(23,41)^ It is important to note that these findings highlight complex relationships; further research is needed to fully understand these associations and their implications for public health interventions in rural India.

Furthermore, while our multivariable analysis showed no statistically significant association between dietary habits and overweight/obesity incidence or remission (Tables 2 and 3), our descriptive analysis (Table 1) showed an increasing prevalence of overweight/obesity among respondents who reported consuming more lentils, fruits, and vegetables. Diets high in plant-based foods like fruits, vegetables and lentils are generally considered to promote weight loss^(42–44)^ as these foods tend to be lower in energy density relative to their volume, promoting satiety and hence less energy intake per meal.^(45)^ However, despite the high intake of lentils, fruits, and vegetables, Indians also consume a large amount of white rice and other refined grain products,^(46)^ along with processed foods which often contain high calories and low nutrients,^(7)^ contributing to an increased prevalence of overweight and obesity. Furthermore, socio-economic status may confound observed prevalence estimates. Further research is needed to understand the complex interplay of factors contributing to overweight/obesity in this population.

Interpreting the study findings should consider a few limitations. First, as assessment of lifestyle risk factors relied on participant recall, findings may be influenced by recall error and social desirability bias. Second, the lack of data on certain potential confounders (for example, sleep quality), may have introduced bias in estimation of relative risks for relationships between other lifestyle and background factors and overweight/obesity incidence and remission. Third, because dietary data were limited to broad food groups, some foods with both obesogenic and anti-obesogenic properties may have been grouped together, potentially affecting the interpretation and translatability of the results. Fourth, although our primary multivariable models include gender as a covariate to account for its potential influence on the observed associations, sample size limitations rendered it infeasible to conduct separate regression models for men and women with adequate statistical power. Finally, because lifestyle risk factors were not measured in the follow-up survey round, inference is limited to effects of factors measured at baseline only, not how changes in those factors over time might influence outcomes; by analysing repeat measurements of BMI, we could nonetheless understand how baseline risk factors are associated with BMI trajectories, which is key toward establishing relationships in observational studies of weight gain and loss.

Overall, this prospective cohort study represents a novel investigation into the long-term dynamics of weight change among rural Indian adults, shedding light on how modifiable lifestyle factors contribute to both the incidence and remission of overweight/obesity. Findings of this study may be used to guide future research on obesity epidemiology, and support design of programmes for mitigating the rapidly escalating burden of overweight/obesity and related impacts on NCD development in rural India. Future research should prioritise more detailed dietary assessment to understand windows for encouraging healthy and locally-acceptable food-based diet modifications. Furthermore, the findings underscore the importance of addressing transportation habits and promoting physical activity. To combat the growing prevalence of overweight/obesity, public health initiatives should prioritise the promotion of active lifestyles by investing in and incentivizing sustainable transportation options, such as expanding cycling infrastructure with dedicated bike lanes and bike-sharing programmes, improving public transportation networks with increased frequency and affordability, and creating pedestrian-friendly environments that prioritise walking and walkability. Additionally, promoting physical activity within the home environment, by encouraging activities such as gardening, vigorous household chores, and active play with children, can be a practical for people who may face social, cultural, or safety-related barriers to exercising outside the home, thereby empowering them to increase their physical activity levels and improve their overall health. However, this study does not suggest limiting outdoor activity. Rather, it highlights that individuals with limited opportunities to exercise outside can engage in household activities to mitigate overweight/obesity. The NPCDCS should be enhanced by incorporating community-level interventions that are both culturally acceptable and feasible within the time constraints of rural Indian men and women.^(47)^ By integrating these culturally sensitive and time-conscious interventions into the NPCDCS, the programme can effectively reach and empower rural communities to adopt healthier lifestyles and reduce their risk of overweight/obesity and associated NCDs.

## Data Availability

The data of this study are available upon reasonable request from the corresponding author and the signing of a data transfer agreement.
